# Supplementary material for: A GPX4-dependent cancer cell state underlies the clear-cell morphology and confers sensitivity to ferroptosis
Source: Nat Commun. 2019 Apr 8;10:1617. doi: 10.1038/s41467-019-09277-9 (PMC6453886; doi:10.1038/s41467-019-09277-9)
Supplement: Supplementary file 1 — Supplementary Information [file 41467_2019_9277_MOESM1_ESM.pdf]

Supplementary Information

A GPX4-dependent cancer cell state underlies the clear-cell morphology and confers sensitivity to ferroptosis

Zou et al.

Contents

Supplementary figures, tables and legends

Supplementary Figure 1. Clear-cell carcinoma cells are sensitive to GPX4 inhibition-induced ferroptosis.

Supplementary Figure 2. Clear-cell carcinoma cells are dependent on GPX4 to evade ferroptosis.

Supplementary Figure 3. Genome-wide CRISPR screen identifies mediators of ferroptosis sensitivity in clear-cell carcinoma cells.

Supplementary Figure 4. The HIF pathway mediates ferroptosis susceptibility in clear-cell carcinomas.

Supplementary Figure 5. HIF-2α selectively enriches polyunsaturated fatty acyl lipids.

Supplementary Figure 6. HILPDA mediates HIF-2α's activity in inducing ferroptosis susceptibility.

Supplementary Figure 7. HILPDA drives PUFA-lipid enrichment in ccRCC cells.

Supplementary Figure 8. Raw immunoblot images.

Supplementary Figure 9. Raw immunoblot images (cont'd).

Supplementary Table 1. Clinical information for the patient-derived primary ccRCC cells.

Supplementary figures, tables and legends

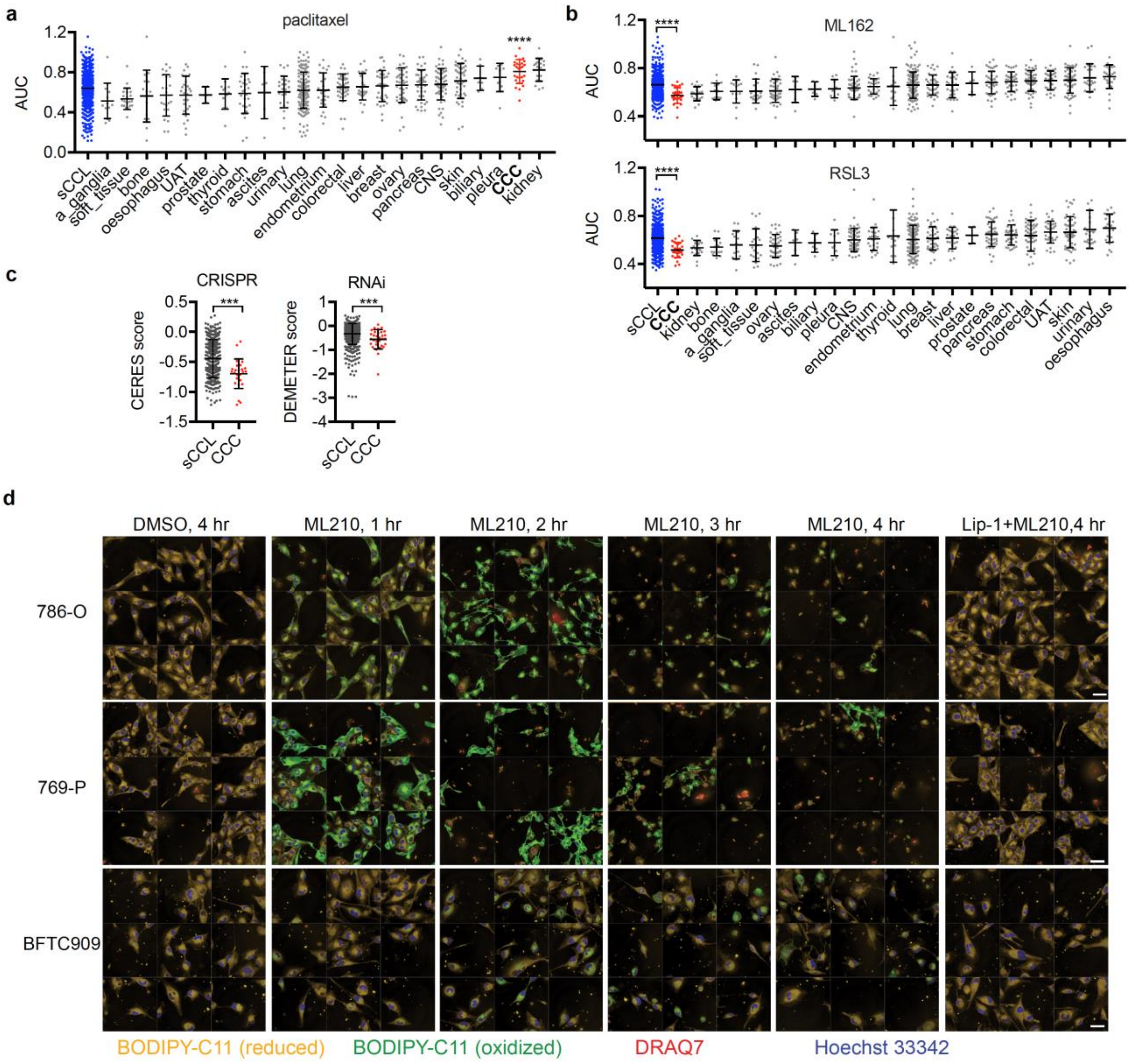

Supplementary Figure 1. Clear-cell carcinoma cells are sensitive to GPX4 inhibition-induced ferroptosis.

a. Scatterplot of AUC value distributions for paclitaxel in solid cancer cell lines (sCCL, blue), clear-cell carcinomas (CCC, red) or cancer cell lines from each specified tissue-of-origin. Larger AUC values indicate lower compound sensitivity and vice versa. Tissue types are ordered by the average AUC values. Abbreviations: CNS, central nervous system; UAT, upper aerodigestive tract; a_ganglia, autonomic ganglia. A Mann-Whitney-Wilcoxon test

was performed between CCCs and other sCCLs. ****, $p<0.0001$. Lines and error bars: mean±s.d.

b. Scatterplot of AUC value distributions for GPX4 inhibitors ML162 and RSL3 in sCCL (blue), CCC (red) or cell lines from each specified tissue-of-origin.

c. Scatterplot of GPX4 dependency scores determined by CRISPR (CERES scores) and RNAi (DEMETER scores) in sCCL (n=396 for CRISPR, and n=603 for RNAi) or CCC (n=25 for CRISPR, and n=31 for RNAi) cell lines in the Cancer Dependency Map (DepMap) database. Mann-Whitney-Wilcoxon, ***, $p<0.001$, lines and error bars: mean±s.d.

d. Fluorescent images of 786-O, 769-P, and BFTC909 cells treated with ML210 and indicated concentrations of vehicle (DMSO) or Lip-1 for the indicated time periods. Nine fields per treatment condition were aligned together for effective visualization. Scale bars: 50 μm.

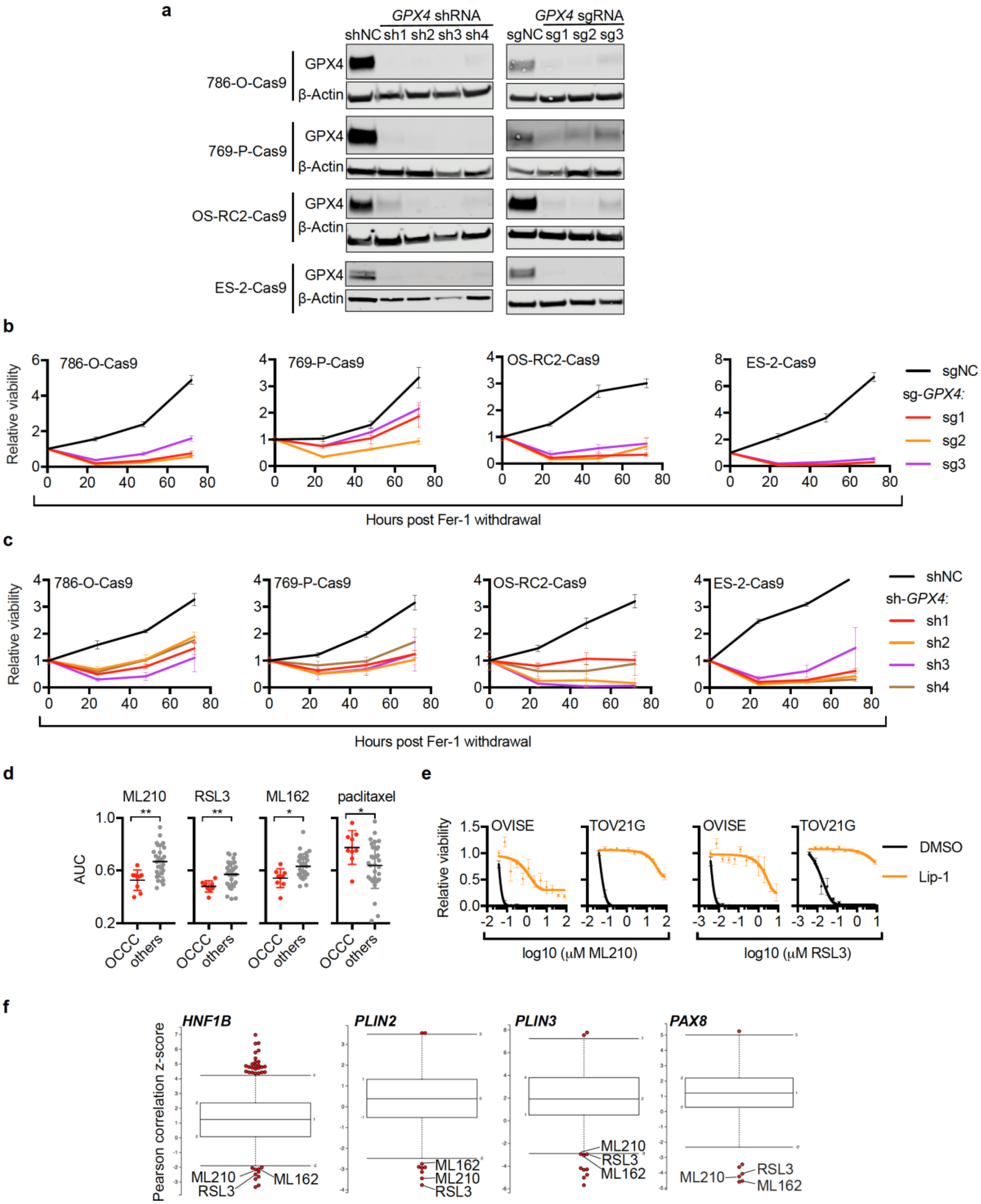
a
GPX4 shRNA
shNC sh1 sh2 sh3 sh4
GPX4 sgRNA
sgNC sg1 sg2 sg3
786-O-Cas9
769-P-Cas9
OS-RC2-Cas9
ES-2-Cas9
GPX4
β-Actin
b
Relative viability
786-O-Cas9
769-P-Cas9
OS-RC2-Cas9
ES-2-Cas9
sgNC
sg-GPX4:
sg1
sg2
sg3
Hours post Fer-1 withdrawal
c
Relative viability
786-O-Cas9
769-P-Cas9
OS-RC2-Cas9
ES-2-Cas9
shNC
sh-GPX4:
sh1
sh2
sh3
sh4
Hours post Fer-1 withdrawal
d
ML210
RSL3
ML162
paclitaxel
AUC
OCCC
others
e
Relative viability
OVISE
TOV21G
DMSO
Lip-1
log10 (μM ML210)
log10 (μM RSL3)
f
HNF1B
PLIN2
PLIN3
PAX8
Pearson correlation z-score
ML210
RSL3
ML162

Supplementary Figure 2. Clear-cell carcinoma cells are dependent on GPX4 to evade ferroptosis.

a. Immunoblot analysis of GPX4 protein levels in ccRCC cell lines 786-O-Cas9, 769-P-Cas9 and OS-RC2-Cas9 and OCCC cell line ES-2-Cas9 expressing control shRNA (shNC) or *GPX4*-targeting shRNAs (left panels), or control sgRNA (sgNC), or GPX4-targeting sgRNAs (right panels). β-Actin was used as a loading control. Representative plot of experiments repeated twice.

b. Viability curves for 786-O-Cas9, 769-P-Cas9, OS-RC2-Cas9 and ES-2-Cas9 cells expressing control shRNA (shNC) or *GPX4*-targeting shRNAs treated with ML210 or RSL3 for 48h. Viability is relative to the DMSO-treated conditions, n=4, error bars: ±s.d.

c. Viability curves for 786-O-Cas9, 769-P-Cas9, OS-RC2-Cas9 and ES-2-Cas9 cells expressing control sgRNA (shNC) or *GPX4*-targeting sgRNAs treated with ML210 or RSL3 for 48h. Viability is relative to the DMSO-treated conditions, n=4, error bars: ±s.d.

d. Scatterplot of AUC value distributions for GPX4 inhibitors ML210, RSL3 and ML162, and chemotherapeutic agent paclitaxel in OCCC (red, n=9) or other ovarian cancer cells (others, gray, n=30). Mann-Whitney-Wilcoxon, *, $p<0.05$, **, $p<0.01$, lines and error bars: mean±s.d.

e. Viability curves for OCCC cell lines OVISE and TOV21G treated with ML210 or RSL3 plus indicated DMSO or liproxstatin-1 (Lip-1).

f. Compound sensitivity- gene-expression correlation analyses in CTRP for CCC markers HNF1B, PLIN2, PLIN3, and PAX8, highlighting that expression levels of these genes are strongly correlated with sensitivity to GPX4 inhibitors ML210, RSL3, and ML162. Negative z-score means high expression is correlated with high sensitivity to compound, and vice versa.

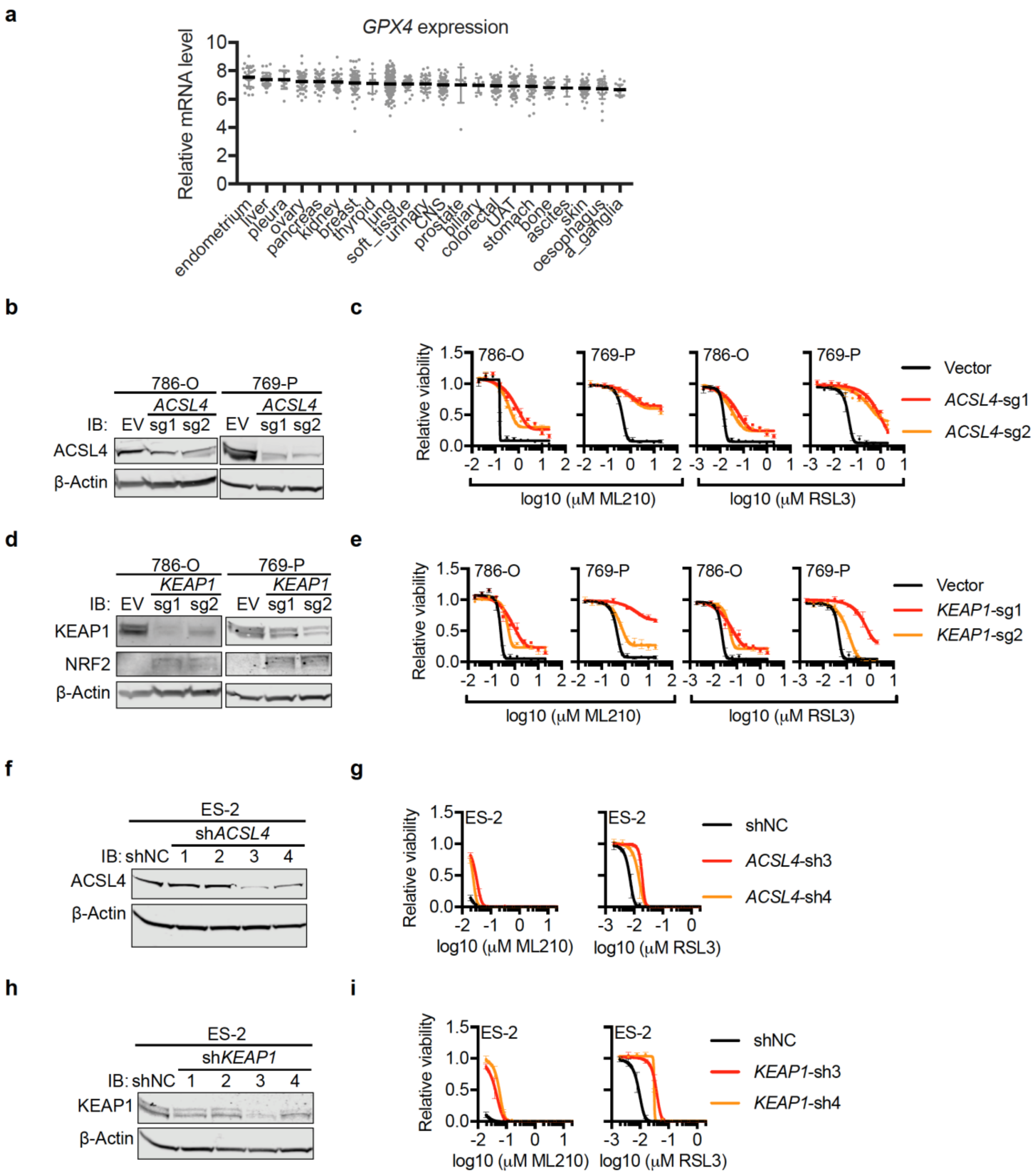

a
GPX4 expression
Relative mRNA level
endometrium
liver
pleura
ovary
pancreas
kidney
breast
thyroid
lung
soft_tissue
urinary
CNS
prostate
biliary
colorectal
UAT
stomach
bone
ascites
skin
oesophagus
a_ganglia
b
786-O
769-P
ACSL4
IB: EV sg1 sg2
ACSL4
β-Actin
c
Relative viability
786-O
769-P
log10 (μM ML210)
log10 (μM RSL3)
Vector
ACSL4-sg1
ACSL4-sg2
d
KEAP1
KEAP1
NRF2
β-Actin
e
Vector
KEAP1-sg1
KEAP1-sg2
f
ES-2
shACSL4
IB: shNC 1 2 3 4
ACSL4
β-Actin
g
shNC
ACSL4-sh3
ACSL4-sh4
h
ES-2
shKEAP1
KEAP1
β-Actin
i
shNC
KEAP1-sh3
KEAP1-sh4

Supplementary Figure 3. Genome-wide CRISPR screen identifies mediators of ferroptosis sensitivity in clear-cell carcinoma cells.

a. Expression of relative GPX4 mRNA levels in cancer cell lines from each indicated tissue-of-origin characterized in CTRP. The cancer types are ordered by the mean of GPX4 expression. Abbreviations: CNS, central nervous system; UAT, upper aerodigestive tract; a_ganglia, autonomic ganglia.

b. Immunoblot analysis of ACSL4 protein levels in vector (EV) and *ACSL4*-targeting sgRNA-expressing 786-O-Cas9 and 769-P-Cas9 cells.

c. Viability curves for WT (Vector) and *ACSL4*-targeting sgRNA-expressing 786-O-Cas9 or 769-P-Cas9 cells under indicated concentrations of ML210 or RSL3.

d. Immunoblot analysis of KEAP1 and NRF2 (NFE2L2) protein levels in WT (Vector) or *KEAP1*-targeting sgRNA-expressing 786-O-Cas9 and 769-P-Cas9 cells.

e. Viability curves for WT (Vector) and *KEAP1*-targeting sgRNA-expressing 786-O-Cas9 or 769-P-Cas9 cells under indicated concentrations of ML210 or RSL3.

f. Immunoblot analysis of ACSL4 protein levels in control (shNC) and *ACSL4*-targeting shRNA- expressing ES-2 cells.

g. Viability curves for control (shNC) and *ACSL4*-targeting shRNA-expressing ES-2 cells under indicated concentrations of ML210 or RSL3. n=4.

h. Immunoblot analysis of KEAP1 protein levels in control (shNC) and *KEAP1*-targeting shRNA- expressing ES-2 cells.

i. Viability curves for control (shNC) and *KEAP1*-targeting shRNA-expressing ES-2 cells under indicated concentrations of ML210 or RSL3. n=4,

Error bars: ±s.d. β-Actin was used as loading controls in immunoblots.

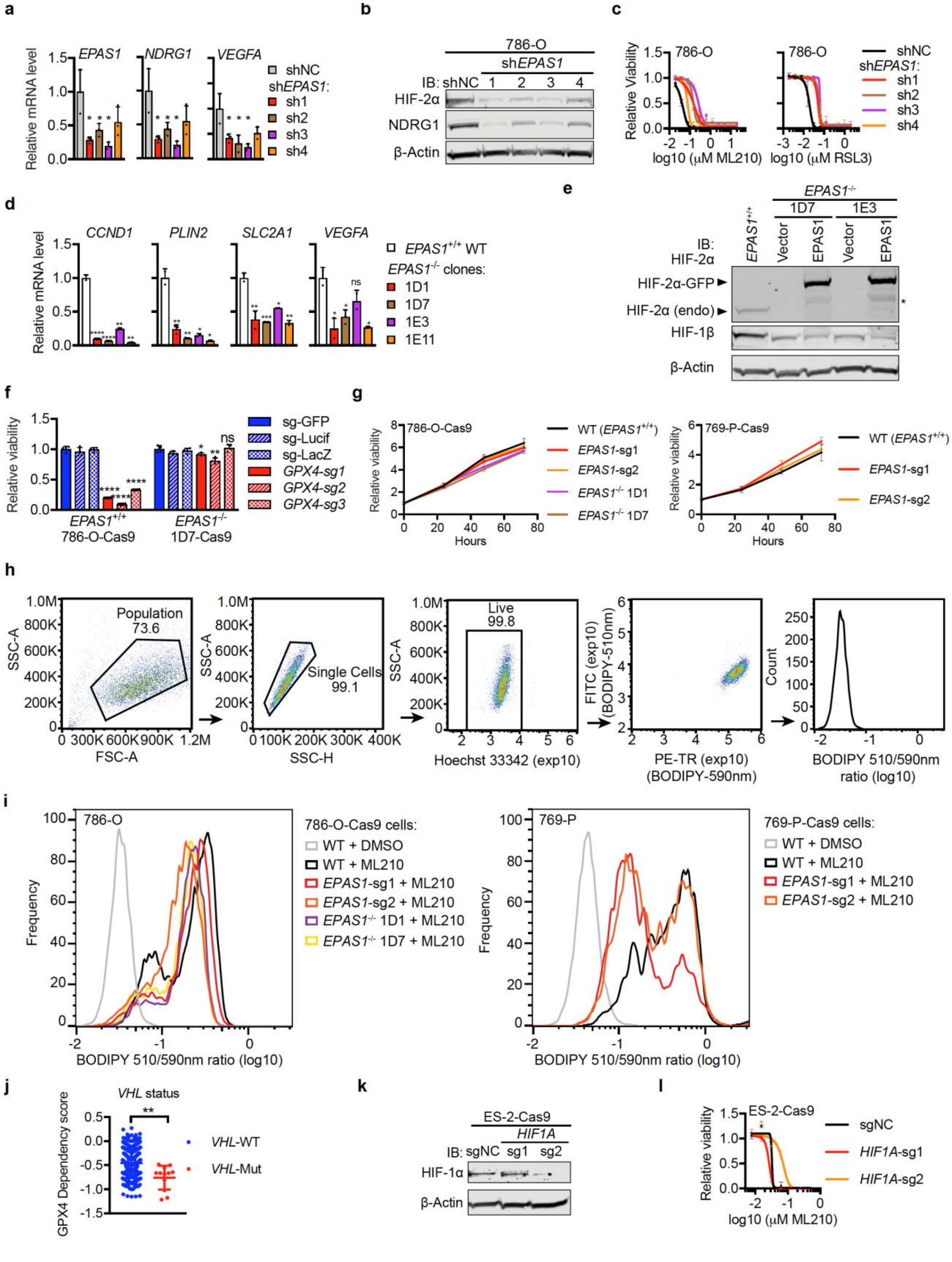
a
EPAS1
NDRG1
VEGFA
Relative mRNA level
shNC
shEPAS1:
sh1
sh2
sh3
sh4
b
786-O
shEPAS1
IB: shNC 1 2 3 4
HIF-2α
NDRG1
β-Actin
c
786-O
Relative Viability
log10 (μM ML210)
log10 (μM RSL3)
shNC
shEPAS1:
sh1
sh2
sh3
sh4
d
CCND1
PLIN2
SLC2A1
VEGFA
Relative mRNA level
EPAS1+/+ WT
EPAS1-/- clones:
1D1
1D7
1E3
1E11
ns
e
EPAS1-/-
1D7
1E3
IB: HIF-2α
EPAS1+/+
Vector
EPAS1
HIF-2α-GFP
HIF-2α (endo)
HIF-1β
β-Actin
f
Relative viability
ns
EPAS1+/+ 786-O-Cas9
EPAS1-/- 1D7-Cas9
sg-GFP
sg-Lucif
sg-LacZ
GPX4-sg1
GPX4-sg2
GPX4-sg3
g
786-O-Cas9
Relative viability
Hours
WT (EPAS1+/+)
EPAS1-sg1
EPAS1-sg2
EPAS1-/- 1D1
EPAS1-/- 1D7
769-P-Cas9
h
Population
73.6
SSC-A
FSC-A
Single Cells
99.1
SSC-H
Live
99.8
Hoechst 33342 (exp10)
FITC (exp10) (BODIPY-510nm)
PE-TR (exp10) (BODIPY-590nm)
Count
BODIPY 510/590nm ratio (log10)
i
786-O
Frequency
BODIPY 510/590nm ratio (log10)
786-O-Cas9 cells:
WT + DMSO
WT + ML210
EPAS1-sg1 + ML210
EPAS1-sg2 + ML210
EPAS1-/- 1D1 + ML210
EPAS1-/- 1D7 + ML210
769-P
769-P-Cas9 cells:
WT + DMSO
WT + ML210
EPAS1-sg1 + ML210
EPAS1-sg2 + ML210
j
VHL status
GPX4 Dependency score
VHL-WT
VHL-Mut
k
ES-2-Cas9
HIF1A
IB: sgNC sg1 sg2
HIF-1α
β-Actin
l
ES-2-Cas9
Relative viability
log10 (μM ML210)
sgNC
HIF1A-sg1
HIF1A-sg2

Supplementary Figure 4. The HIF pathway mediates ferroptosis susceptibility in clear-cell carcinomas.

a. qRT-PCR analyses of mRNA levels of *EPAS1* and HIF-2α target genes *NDRG1* and *VEGFA* in 786-O cells expressing shNC or *EPAS1*-targeting shRNAs. *GAPDH* was used as an internal control. n=3. Student's T-test, *, p<0.05.

b. Immunoblot analysis of NDRG1 and HIF-2α protein expression in 786-O cells expressing shNC or *EPAS1*-targeting shRNAs.

c. Viability curves for the indicated cells treated with ML210 or RSL3. n=4. Representative plot of experiments repeated three times.

d. qRT-PCR analyses of mRNA levels of HIF-2α target genes *CCND1*, *PLIN2*, *SLC2A1* (GLUT1), and *VEGFA* in 786-O WT or the indicated *EPAS1*$^{-/-}$ clones. *B2M* was used as an internal control. n=2 or 3. Two-tailed student's T-test, *, p<0.05, **, p<0.01, ***, p<0.001, ****, p<0.0001, ns, not significant.

e. Immunoblot analysis of HIF-2α and HIF-1β protein levels in wildtype (*EPAS1*$^{+/+}$), two *EPAS1*$^{-/-}$ single-cell clones 1D7 and 1E3, each expressing an empty vector or *EPAS1*-GFP (*EPAS1*) construct. Asterisk indicates a non-specific band.

f. Relative viability of *EPAS1*$^{+/+}$ 786-O-Cas9 and *EPAS1*$^{-/-}$ 1D7-Cas9 cells transduced with control (sg-*GFP*, sg-*Firefly Luciferase*, or sg-*LacZ*) or *GPX4*-targeting sgRNAs at day 7 post-infection. n=4. Two-tailed student's t-test was performed between each *GPX4*-targeting sgRNA and the sg-*GFP* condition. *, p<0.05, **, p<0.01, ****, p<0.0001, ns, not significant.

g. Viability time course for 786-O-Cas9 (left panel) cells expressing control sgRNAs (WT) or *EPAS1*-targeting sgRNAs, and *EPAS1*$^{-/-}$ clones 1D1 and 1D7, and 769-P-Cas9 (right panel) cells expressing control sgRNAs (WT) or *EPAS1*-targeting sgRNAs. n=8.

h. Example gating strategy for flow cytometry analysis of BODIPY-C11 oxidation in cells.

i. Flow cytometry analyses of lipid peroxidation levels in the indicated cells.

j. Scatterplot showing the GPX4 dependency scores (CERES) by CRISPR in cancer cell lines possessing wildtype (WT, n=421; blue) or mutant *VHL* (n=12; red) in the DepMap database. Mann-Whitney-Wilcoxon test, **, p < 0.01.

k. Immunoblot showing the HIF-1α protein levels in control (sgNC) or *HIF1A*-targeting sgRNA-expressing ES-2-Cas9 cells.

l. Viability curves of indicated cells treated with ML210. n=4. Representative plot of experiments repeated three times.

Error bars: ±s.d. β-Actin was used as loading controls in immunoblots.

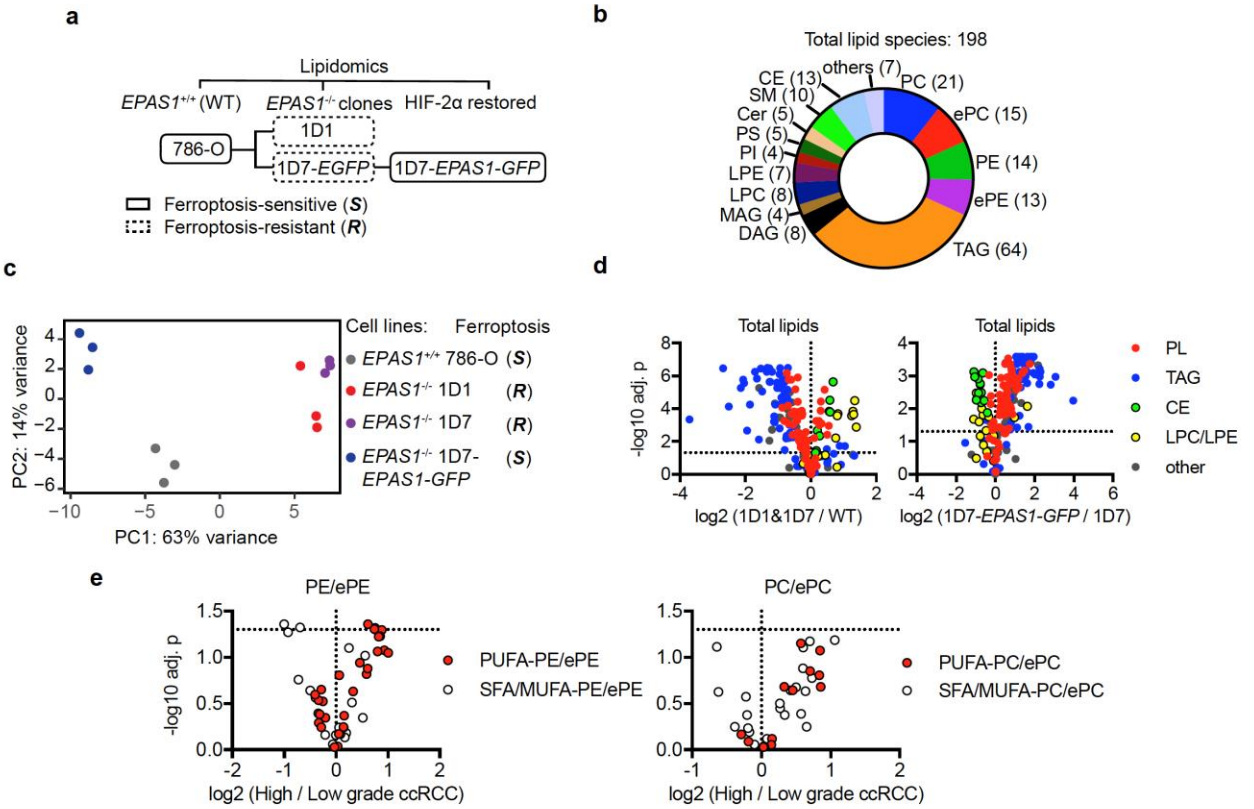

Supplementary Figure 5. HIF-2α selectively enriches polyunsaturated fatty acyl lipids.

a. Scheme summarizing the lipidomics experiment with 786-O WT, *EPAS1*$^{-/-}$ derivative clones 1D1 and 1D7 (1D7-*EGFP*), and 1D7-*EPAS1-GFP* cells. Three biological replicates were analyzed in each condition. (***S***), ferroptosis-sensitive; (***R***), ferroptosis-resistant.

b. Pie chart summarizing the number of lipid species from each class detected in the lipidomic profiling of 786-O WT cells. Abbreviations: CE, cholesterol ester; Cer, ceramide; MAG, monoacylglycerol; DAG, diacylglycerol; TAG, triacylglycerol; LPC, lysophosphatidylcholine; LPE, lysophosphatidylethanolamine; PC, phosphatidylcholine; PE, phosphatidylethanolamine; ePC, (vinyl ether-linked) PC-plasmalogen; ePE, (vinyl ether-linked) PE-plasmalogen; PI, phosphatidylinositol; PS, phosphatidylserine; SM, sphingomyelin.

c. Principal component plot of the lipidomic profiles for the indicated cell lines.

d. Volcano plot highlighting the changes of the indicated lipid classes between the indicated groups. Abbreviations are the same as in panel **b**.

e. Volcano plots showing changes in each PE/ePE species grouped as PUFA-PE/ePEs (red fill) and SFA/MUFA-PE/ePEs (white fill) between high-grade (grade III/IV, n=18) and low-grade (grade I/II, n=16) ccRCC tumor samples.

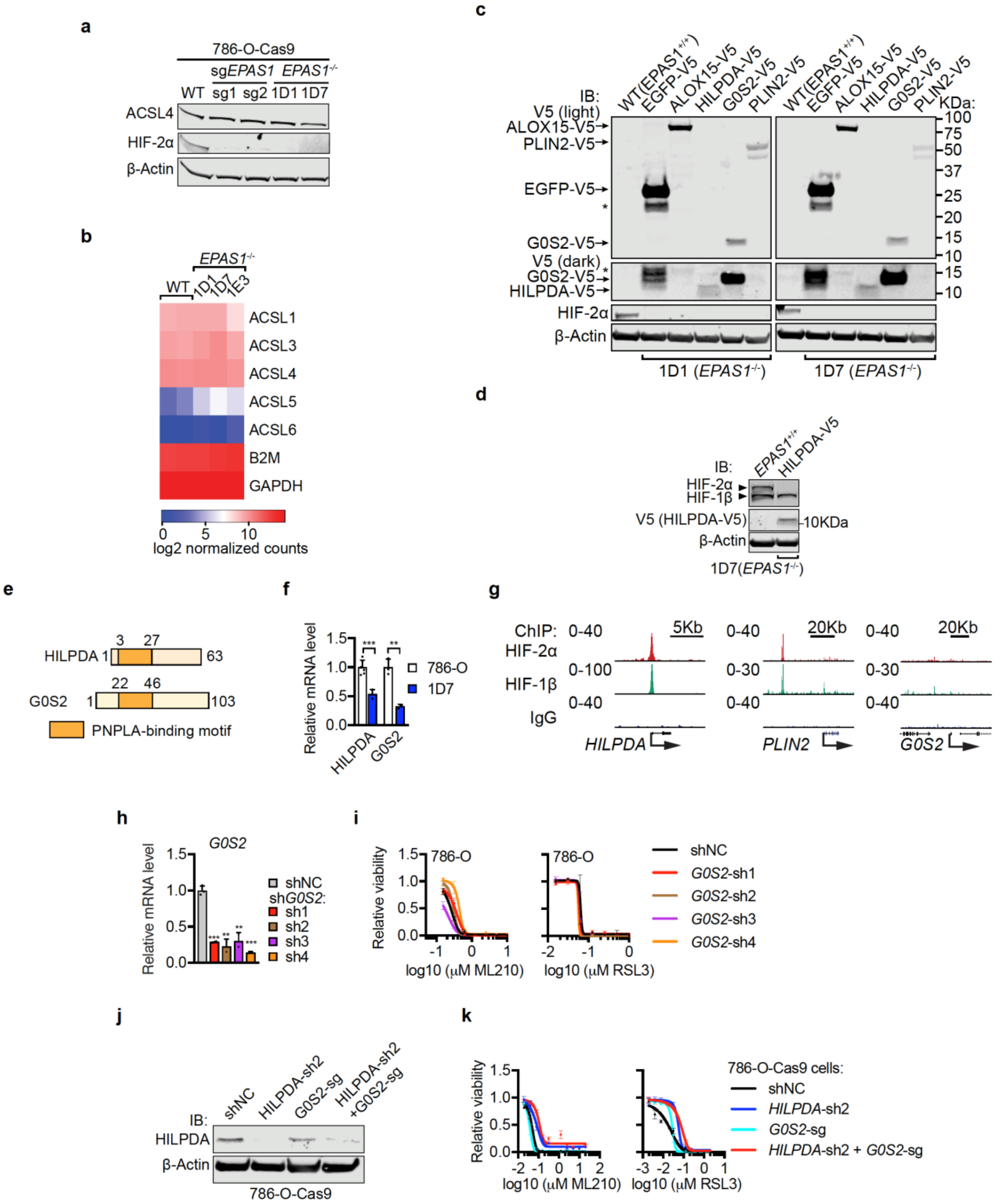
a
786-O-Cas9
sgEPAS1
EPAS1-/-
WT sg1 sg2 1D1 1D7
ACSL4
HIF-2α
β-Actin
b
EPAS1-/-
WT 1D1 1D7 1E3
ACSL1
ACSL3
ACSL4
ACSL5
ACSL6
B2M
GAPDH
0 5 10
log2 normalized counts
c
IB:
V5 (light)
WT(EPAS1+/+) EGFP-V5 ALOX15-V5 HILPDA-V5 G0S2-V5 PLIN2-V5
KDa:
ALOX15-V5
PLIN2-V5
EGFP-V5
G0S2-V5
V5 (dark)
G0S2-V5
HILPDA-V5
HIF-2α
β-Actin
100 75 50 37 25 20 15 10
15 10
1D1 (EPAS1-/-)
1D7 (EPAS1-/-)
d
IB:
EPAS1+/+ HILPDA-V5
HIF-2α
HIF-1β
V5 (HILPDA-V5)
10KDa
β-Actin
1D7(EPAS1-/-)
e
HILPDA 1 3 27 63
G0S2 1 22 46 103
PNPLA-binding motif
f
Relative mRNA level
786-O
1D7
HILPDA G0S2
g
ChIP:
HIF-2α
HIF-1β
IgG
0-40 0-100 0-40
5Kb
0-40 0-30 0-40
20Kb
HILPDA
PLIN2
G0S2
h
G0S2
Relative mRNA level
shNC
shG0S2:
sh1
sh2
sh3
sh4
i
786-O
Relative viability
log10 (μM ML210)
log10 (μM RSL3)
shNC
G0S2-sh1
G0S2-sh2
G0S2-sh3
G0S2-sh4
j
IB:
shNC HILPDA-sh2 G0S2-sg HILPDA-sh2 +G0S2-sg
HILPDA
β-Actin
786-O-Cas9
k
Relative viability
log10 (μM ML210)
log10 (μM RSL3)
786-O-Cas9 cells:
shNC
HILPDA-sh2
G0S2-sg
HILPDA-sh2 + G0S2-sg

Supplementary Figure 6. HILPDA mediates HIF-2α's activity in inducing ferroptosis susceptibility.

a. Immunoblot analysis of ACSL4 protein levels in 786-O-Cas9 cells expressing control sgRNAs (WT) or *EPAS1*-targeting sgRNAs, and *EPAS1*$^{-/-}$ clones 1D1 and 1D7.

b. Heatmap showing expression levels of ACSL family members in the RNA-Seq analysis of WT 786-O cells and three *EPAS*$^{-/-}$ clones (1D1, 1D7 and 1E3). B2M and GAPDH are house-keeping genes included as controls.

c. Immunoblot analysis of protein levels of HIF-2α, and V5 tagged EGFP, ALOX15, HILPDA, G0S2, and PLIN2 in *EPAS*$^{-/-}$ 1D1 (left) and 1D7 (right) cells. Two exposure levels were presented for the low molecular weight part of the membranes. Asterisks indicate non-specific bands. Representative plot of experiments repeated three times.

d. Immunoblot analysis of protein levels of HIF-2α, HIF-1β, and V5 tagged HILPDA in *EPAS*$^{-/-}$ 1D7 cells to verify the lowest molecular weight results in panel **c**.

e. Scheme summarizing the secondary protein structures of HILPDA and G0S2, highlighting the shared PNPLA-inhibitory motif (dark orange). Numbers indicate amino acid residues.

f. qRT-PCR analysis of mRNA levels of HILPDA and G0S2 in 786-O-WT and *EPAS1*$^{-/-}$ 1D7 cells. Two-tailed student's T-test, **, $p<0.01$, ***, $p<0.001$. B2M was used as an internal control. n=3. Representative plot of experiments repeated three times.

g. Gene-track view for the ChIP-Seq analysis of HIF-2α and HIF-1β in 786-O cells (GSE34871) at the *HILPDA, PLIN2*, and *G0S2* loci. Y-axis: reads per million reads (RPM).

h. qRT-PCR analysis of *G0S2* mRNA levels in 786-O cells expressing shNC or sh*G0S2*s. B2M was used as an internal control. n=3. Two-tailed student's T-test. *, $p< 0.05$.

i. Viability curves for the indicated cells treated with ML210 or RSL3. n=4.

j. Immunoblot analysis of HILPDA protein levels in 786-O-Cas9 cells expressing control shRNA (shNC), *HILPDA*-targeting shRNA, G0S2-targeting sgRNA or *HILPDA*-targeting shRNA and G0S2-targeting sgRNA together. **(**G0S2 protein level was below the detection limit of multiple G0S2-targeting antibodies.**)** Representative plot of experiments repeated twice.

k. Viability curves for the indicated cells treated with ML210 or RSL3. n=4.

Error bars: ±s.d. β-Actin was used as loading controls in immunoblots.

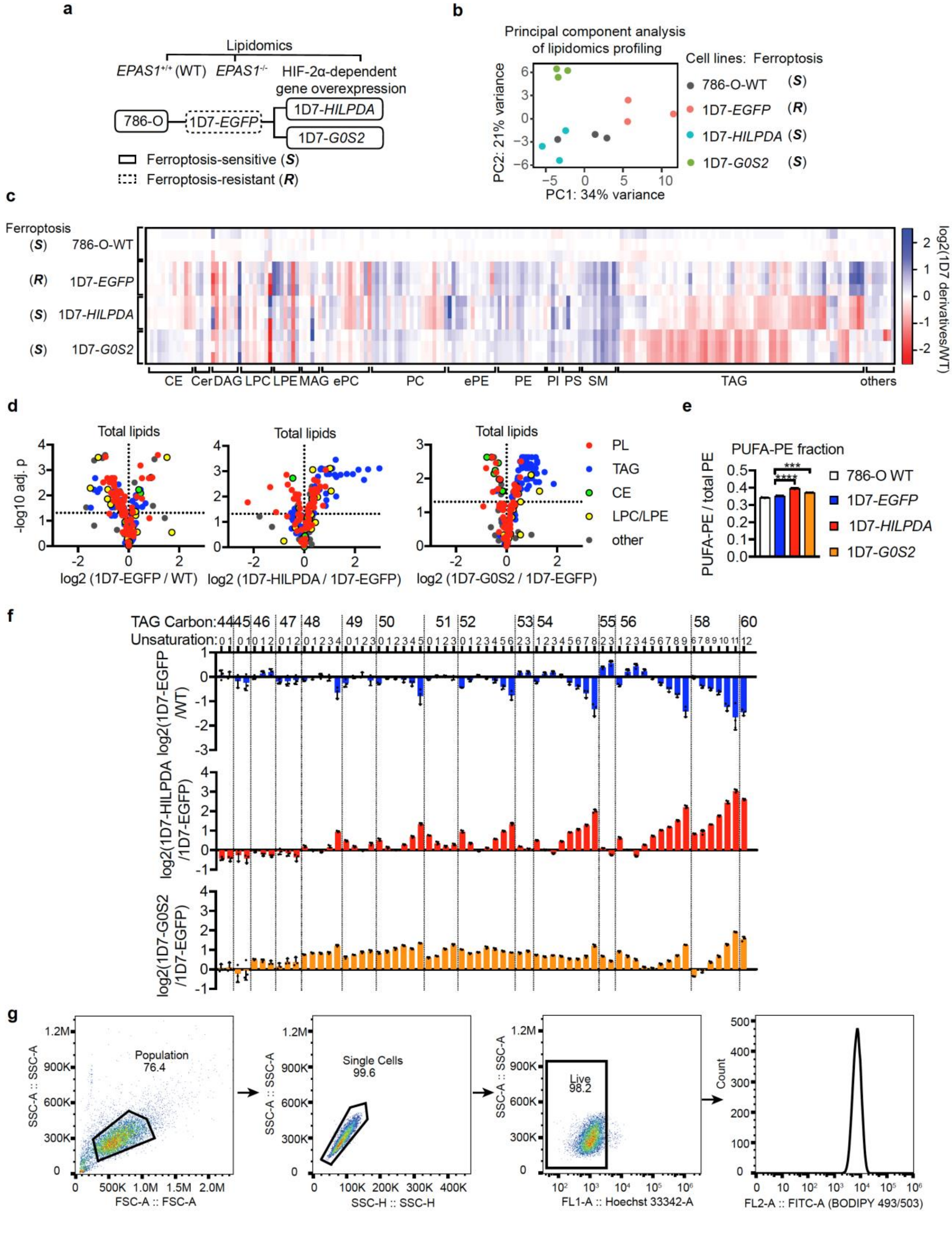

a
Lipidomics
EPAS1+/+ (WT)
EPAS1-/-
HIF-2α-dependent gene overexpression
786-O
1D7-EGFP
1D7-HILPDA
1D7-G0S2
Ferroptosis-sensitive (S)
Ferroptosis-resistant (R)
b
Principal component analysis of lipidomics profiling
Cell lines: Ferroptosis
786-O-WT (S)
1D7-EGFP (R)
1D7-HILPDA (S)
1D7-G0S2 (S)
PC2: 21% variance
PC1: 34% variance
c
Ferroptosis
(S) 786-O-WT
(R) 1D7-EGFP
(S) 1D7-HILPDA
(S) 1D7-G0S2
CE Cer DAG LPC LPE MAG ePC PC ePE PE PI PS SM TAG others
log2(1D7 derivatives/WT)
d
Total lipids
-log10 adj. p
log2 (1D7-EGFP / WT)
log2 (1D7-HILPDA / 1D7-EGFP)
log2 (1D7-G0S2 / 1D7-EGFP)
PL
TAG
CE
LPC/LPE
other
e
PUFA-PE fraction
PUFA-PE / total PE
786-O WT
1D7-EGFP
1D7-HILPDA
1D7-G0S2
f
TAG Carbon:
Unsaturation:
log2(1D7-EGFP /WT)
log2(1D7-HILPDA /1D7-EGFP)
log2(1D7-G0S2 /1D7-EGFP)
g
Population 76.4
Single Cells 99.6
Live 98.2
SSC-A :: SSC-A
FSC-A :: FSC-A
SSC-H :: SSC-H
FL1-A :: Hoechst 33342-A
Count
FL2-A :: FITC-A (BODIPY 493/503)

Supplementary Figure 7. HILPDA drives PUFA-lipid enrichment in ccRCC cells.

a. Scheme summarizing the lipidomic profiling experiments for 786-O and *EPAS1*$^{-/-}$ 1D7 cells expressing EGFP, HILPDA, or G0S2. Three biological replicates were analyzed in each condition. (***S***), ferroptosis-sensitive; (***R***), ferroptosis-resistant.

b. Principal component plot of lipidomic profiles for the indicated cell lines.

c. Heatmap representing the relative lipid abundances in the indicated cell lines. The lipid ratios between 1D7 derivatives and 786-O WT cells were log2 transformed and plotted. Lipid class abbreviations are the same as **Fig. 3a**.

d. Volcano plots highlighting the changes of the indicated lipid classes between the indicated groups. Lipid class abbreviations are the same as **Fig. 3a**.

e. The ratio between PUFA-PE/ePE and total PE/ePE in the indicated cell lines. Two-tailed student's T-test. ***, $p < 0.001$, ****, $p < 0.0001$.

f. Bar graph showing the relative abundances of each TAG species between the cell lines indicated. The lipid ratios between 1D7 derivatives and 786-O WT cells were log2 transformed and plotted. n=3, error bars: ±s.d.

g. Example gating strategy for flow cytometry analysis of lipid droplet contents by BODIPY-493/503 in *EPAS1*$^{-/-}$ 786-O and derivative cells.

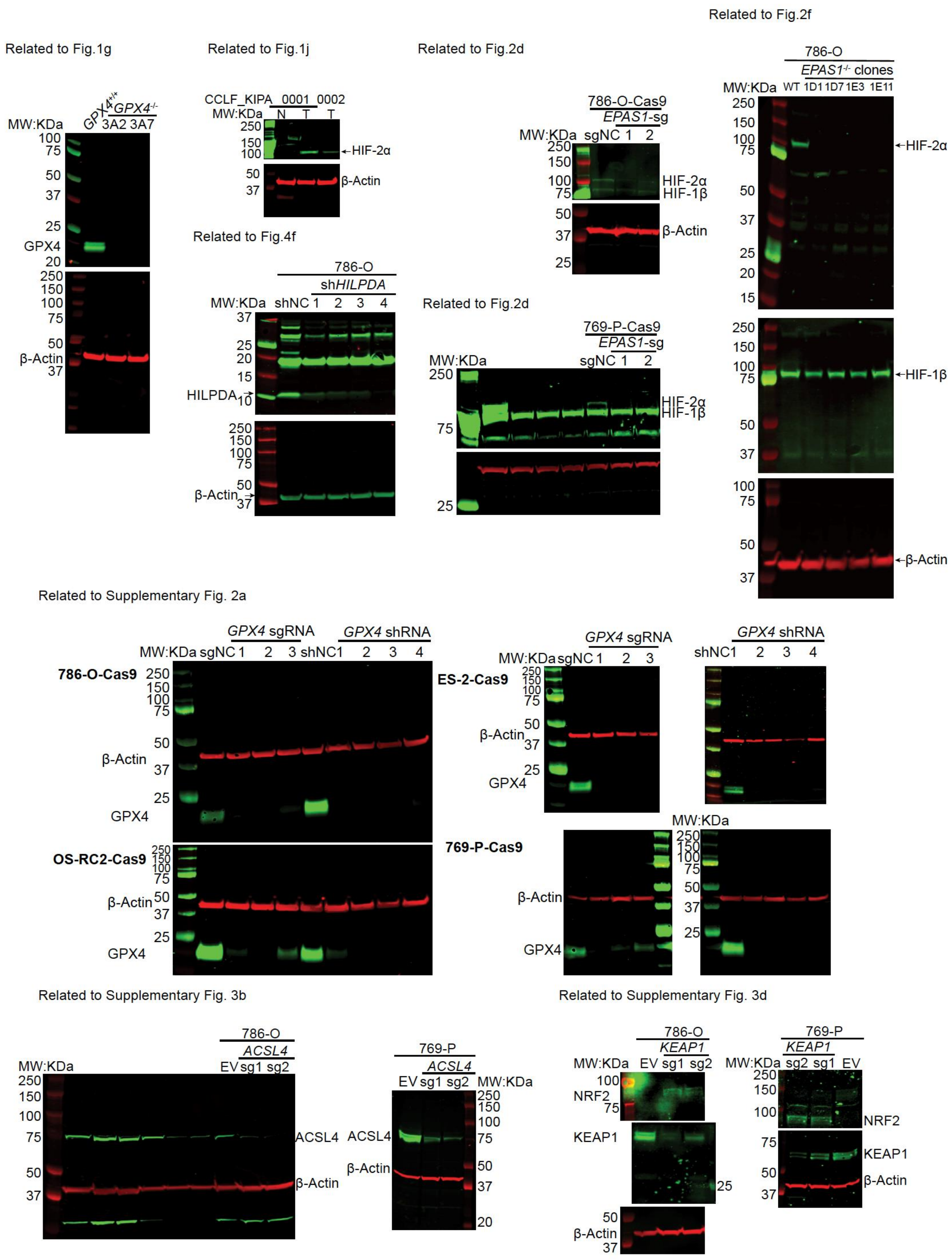

Supplementary Figure 8. Raw immunoblot images.

Related to Supplementary Fig. 3f

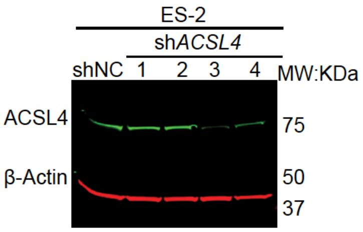

Related to Supplementary Fig. 3h

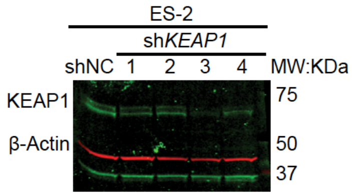

Related to Supplementary Fig. 4b

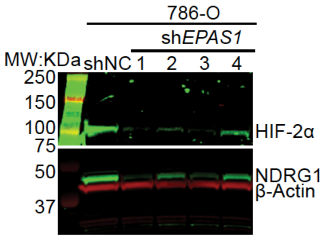

Related to Supplementary Fig. 4e

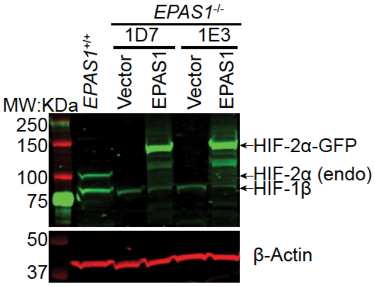

Related to Supplementary Fig. 4k

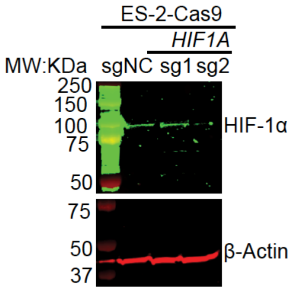

Related to Supplementary Fig. 6a

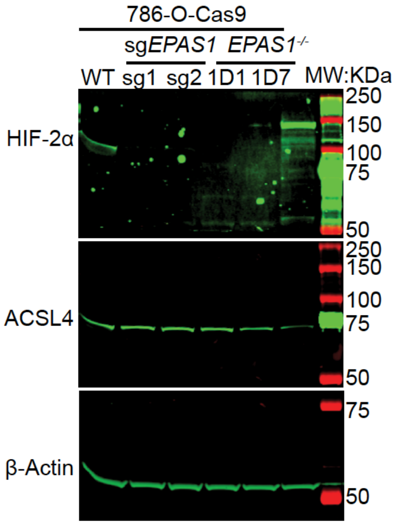

Related to Supplementary Fig. 6d

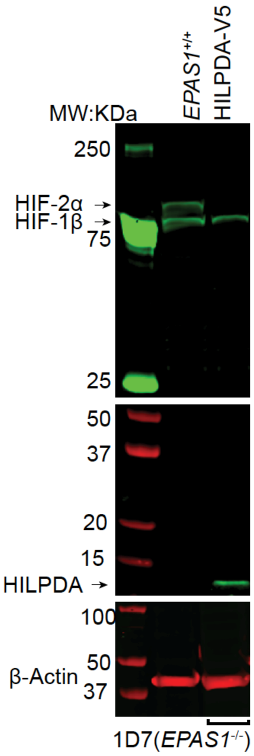

Related to Supplementary Fig. 6j

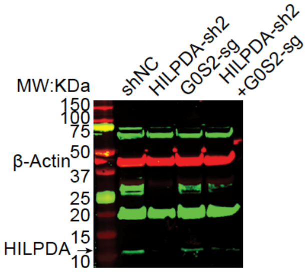

Related to Supplementary Fig. 6c

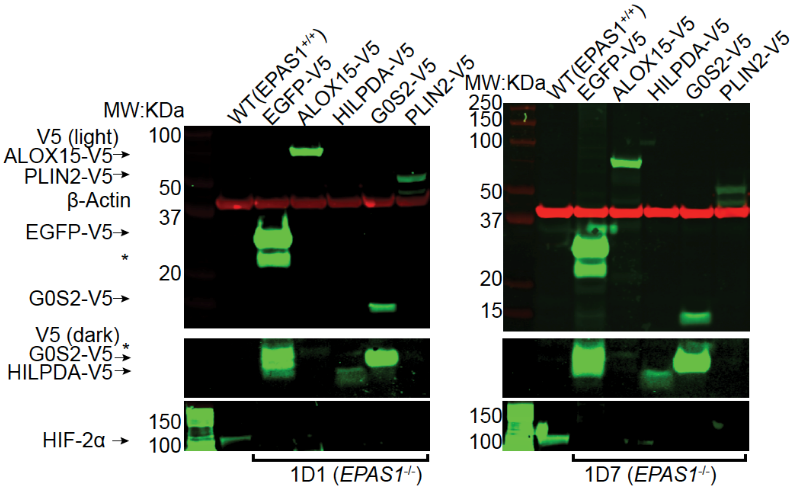

Supplementary Figure 9. Raw immunoblot images (cont'd).

Population characteristics	Age	Gender	Genotypic info	Diagnosis Present/previous	Treatment history	Comments
CCLF_KIPA_0001	66	F	Oncopanel analyzed	renal cell carcinoma, clear cell, pT1bN0M0, Grade II	observation for RCC tamoxifen for breast cancer	also had breast cancer
CCLF_KIPA_0002	62	M	Oncopanel not analyzed	renal cell carcinoma, clear cell, pT3aN0, Grade III	observation for RCC, no systemic therapy local stereotactic radiation therapy for a metastatic lesion	

Supplementary Table 1. Clinical information for the patient-derived primary ccRCC cells.
