# Supplementary material for: A GPX4-dependent cancer cell state underlies the clear-cell morphology and confers sensitivity to ferroptosis
Source: Nat Commun. 2019 Apr 8;10:1617. doi: 10.1038/s41467-019-09277-9 (PMC6453886; doi:10.1038/s41467-019-09277-9)
Supplement: Supplementary file 3 — Description of Additional Supplementary Files [file 41467_2019_9277_MOESM3_ESM.docx]

**Description of Additional Supplementary Files**

File Name: Supplementary Data 1

Description: CRISPR screen analysis of day 4 ML210 vs DMSO-treated conditions.

File Name: Supplementary Data 2

Description: CRISPR screen analysis of day 6 ML210 vs DMSO-treated conditions.

File Name: Supplementary Data 3

Description: CRISPR screen analysis of day 8 ML210 vs DMSO-treated conditions.

File Name: Supplementary Data 4

Description: Lipidomic profiling of 786-O WT, *EPAS1* knockout and *EPAS1-GFP* rescue cells.

File Name: Supplementary Data 5

Description: HILIC-negative lipidomic profiling of free fatty acids in 786-O WT, *EPAS1* knockout and *EPAS1-GFP* rescue cells.

File Name: Supplementary Data 6

Description: ORFs used in the cDNA screen experiment in *EPAS1*^-/-^ 786-O cells.

File Name: Supplementary Data 7

Description: Lipidomic profiling of 786-O WT and EPAS1 knockout cells expressing EGFP, HILPDA or G0S2.

File Name: Supplementary Data 8

Description: qRT-PCR primer sequences used for gene expression analysis.

File Name: Supplementary Data 9

Description: sgRNA sequences used in CRISPR/Cas9-mediated genome editing.

File Name: Supplementary Data 10

Description: shRNA sequences used in gene knockdown.
